# Isolated Neurosarcoidosis Presenting as Chronic Progressive Pachymeningitis

**DOI:** 10.1155/2023/2140740

**Published:** 2023-03-09

**Authors:** Joshua Abata, Danielle Bazer, Nicholas Koroneos, Olga Syritsyna

**Affiliations:** Stony Brook University Hospital, 1101 Nicolls Rd, Stony Brook, NY 11794, USA

## Abstract

Neurologic manifestations of sarcoidosis are rare, and even rarer still are cases of isolated neurosarcoidosis. The clinical presentation of isolated neurosarcoidosis can be highly variable, and diagnosis is particularly challenging, the gold standard being tissue biopsy. We describe a patient with a history of atypical parkinsonian syndrome and chronic right frontal lobe infarct who developed weakness, imbalance, and gait disequilibrium in 2008, with magnetic resonance imaging at that time showing leptomeningeal and nodular enhancements in the bilateral frontal and parietal lobes. The patient had an extensive negative workup in 2010 but ultimately did not receive a definitive diagnosis with a tissue biopsy until 2020. The patient also notably failed a 3-month course of steroids after his biopsy due to a lack of symptomatic improvement. This case highlights the clinical variability and diagnostic difficulties of isolated neurosarcoidosis. We also highlight that our patient did not have any symptomatic improvement on steroids, which do typically provide some relief for patients.

## 1. Introduction

Sarcoidosis is a multisystem disorder characterized by noncaseating granulomas. Neurologic complications of sarcoidosis can occur in approximately 5–26% of sarcoidosis cases [1, 2]. Isolated neurosarcoidosis is an even rarer subset of neurosarcoidosis, accounting for only 11% of neurosarcoidosis in one case series [3]. The clinical presentation of neurosarcoidosis can be highly variable, but common manifestations can include cranial neuropathy, peripheral neuropathy, mononeuropathy, myopathy, psychiatric disorders, and cerebellar ataxia, and compared to systemic neurosarcoidosis, isolated neurosarcoidosis seems to more commonly present with headache, hemiparesis, and radiculopathy [2, 3].

## 2. Case Presentation

A 73-year-old wheelchair-bound male, with a history of chronic right frontal lobe infarct with residual left-sided weakness, atypical parkinsonian syndrome, three-vessel coronary artery disease, non-ST segment elevation myocardial infarction, hypertension, hyperlipidemia, and neurogenic bladder, presented with weakness, imbalance, and gait disequilibrium beginning in 2008. Brain magnetic resonance imaging in January 2008 was remarkable for leptomeningeal and nodular enhancement in both cerebral hemispheres involving the frontal and parietal lobes, suggestive of frontoparietal pachymeningitis. He was also found to have midcervical myelopathy and underwent cervical spine laminectomy in 2009. There was some question of a dural fistula of the superior sagittal sinus, but a cerebral angiogram was negative for vascular malformation. An outside lumbar puncture in 2008 was reportedly normal. In 2010, the patient had an extensive workup including a lumbar puncture and serology, which was negative. Cerebrospinal fluid studies included Gram stain, fungal culture, acid-fast stain, tuberculosis culture, malignant cell cytology, serum protein electrophoresis, immunoglobulin G index, oligoclonal bands, myelin basic protein, cryptococcal antigen, and viral panel (herpes simplex virus, arbovirus, California encephalitis virus, Saint Louis encephalitis virus, western equine encephalitis virus, eastern equine encephalitis virus, and West Nile virus). Serology studies included the complete blood count with differential, angiotensin-converting enzymes, immunoglobulin G typing, erythrocyte sedimentation rates, C-reactive protein, antinuclear antibodies, cytoplasmic antineutrophil cytoplasmic antibodies, perinuclear antineutrophil cytoplasmic antibodies, atypical anti-neutrophil cytoplasmic antibodies, Lyme titers, rheumatoid factor, rapid plasma reagin, venereal disease research laboratory tests, viral cultures, and Sjogren's syndrome antibody testing. 24-hour urinary copper was also normal. Electromyography showed the right carpal tunnel and fifth lumbar radiculopathy. The patient declined a meningeal biopsy at that time and was given the presumptive diagnosis of idiopathic pachymeningitis.

Following this workup in 2010, the patient had interval brain magnetic resonance imaging which was relatively stable but with some fluctuations in the leptomeningeal enhancements which, according to his daughter, appeared to correlate with clinical fluctuations in weakness and mobility. The patient's functional status gradually worsened from 2010 to 2012, as he was no longer able to drive or work in 2010. In 2011, he began having difficulty walking without a handrail and had intermittent falls once per year. He was treated at an outside hospital for a right frontal lobe stroke with residual left-sided weakness in 2012. Magnetic resonance imaging on presentation to our hospital in 2020 showed chronic deep white matter infarct in the right frontal lobe. The patient was also diagnosed with an atypical parkinsonian syndrome in 2013 and had follow-up at our Parkinson's and Movement Disorders Center through 2020, due to his neurologic examination showing flat affect, fluent but dysarthric speech, ideomotor apraxia, moderate hypomimia, mildly diminished downward ocular saccades, neck rigidity, asymmetric left-predominant bradykinesia, and postural instability. The patient was trialed on multiple treatments, including pramipexole, carbidopa-levodopa, and ropinirole without much improvement in his parkinsonian symptoms. He ultimately became wheelchair-bound in 2019.

**Figure 1:**
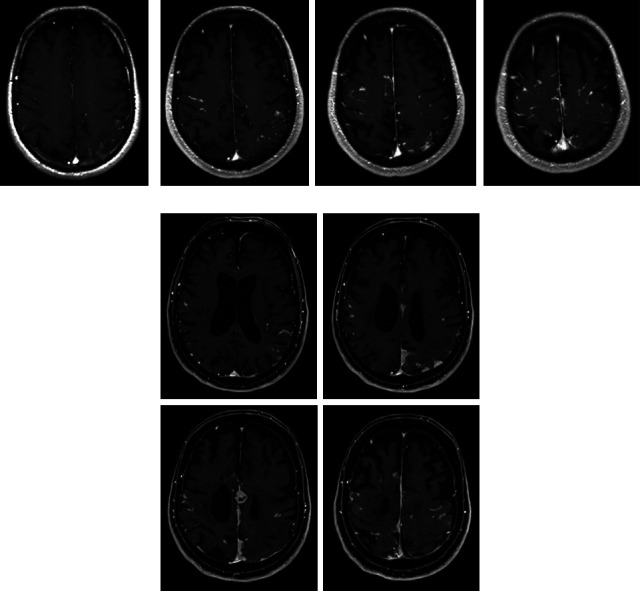
Axial T1 (longitudinal relaxation time) magnetic resonance imaging of the brain with contrast in (a) January 2008, (b) April 2010, and (c) January 2020, showing progression of multiple heterogeneously enhancing dural base and leptomeningeal lesions.

During hospitalization for sepsis and cardiogenic shock in January 2020, repeat brain magnetic resonance imaging showed further interval increase in the size and number of multiple heterogeneously enhancing dural base and leptomeningeal lesions compared to brain magnetic resonance imaging performed in October 2019 at an outside hospital (Figure 1). Of note, outside October 2019 imaging also showed an interval increase in leptomeningeal nodular enhancement compared to prior imaging in 2017. The lumbar puncture was unremarkable. Magnetic resonance imaging of the cervical and thoracic spine showed only some age-related changes. Chest computerized tomography did not show any abnormalities related to possible sarcoidosis. The patient subsequently underwent a brain biopsy in September 2020, which showed granulomatous pachymeningitis without necrosis, suggestive of neurosarcoidosis. He then received treatment with 3 months of prednisone 60 mg daily from October 2020 to January 2021, discontinued after lack of symptomatic improvement in his weakness and mobility in addition to development of leg swelling. Further treatment with dexamethasone and disease-modifying therapy was offered. However, after discussion, the patient, his family, and the care team ultimately agreed not to proceed given his comorbidities and age.

## 3. Discussion

This case illustrates the limitations of current diagnostic modalities for isolated neurosarcoidosis, as the patient underwent an extensive negative workup before arriving at a diagnosis with a tissue biopsy. The gold standard diagnostic test for both systemic and isolated neurosarcoidosis is a tissue biopsy, but if biopsy is not feasible, systemic neurosarcoidosis can be diagnosed with evidence of a neuroinflammatory process coupled with evidence of sarcoidosis outside the nervous system [4]. The latter diagnostic option is of course moot for isolated neurosarcoidosis. Magnetic resonance imaging is the imaging modality of choice for neurosarcoidosis, which did show evidence of meningeal lesions in our patient [5]. Computerized tomography can also be used but is less sensitive and specific than magnetic resonance imaging [6]. A highly sensitive marker for a neuroinflammatory process is enhancement on contrast-enhanced magnetic resonance imaging, which indicates blood-brain-barrier breakdown, which was present in this patient [7]. The cerebrospinal fluid findings including lymphocytic pleocytosis, elevated protein, low glucose, and oligoclonal bands can also indicate a neuroinflammatory process [7]. It is also worth mentioning that there are currently no reliable biomarkers to diagnose or monitor neurosarcoidosis [7]. The patient did notably have a negative serum angiotensin-converting enzyme as a part of his workup. Though it is often included in sarcoidosis workups, it is poorly diagnostic, and the test's sensitivity can range from 41 to 89% [8].

This patient's workup was primarily notable for enhancing lesions from his first magnetic resonance imaging in 2008, which was indicative of a neuroinflammatory process, but his workup was negative for sarcoidosis outside the nervous system. Ultimately, he declined biopsy until 12 years after initial detection of his meningeal lesions. This case underlines the difficulties associated with diagnosing isolated neurosarcoidosis without a tissue biopsy.

It is also worth discussing the patient's lack of response to steroids. The general treatment algorithm for neurosarcoidosis is to start with steroids, which can show improvement/stability in 40–82% of patients [9]. Second-line treatment is typically an immunosuppressant such as hydroxychloroquine, azathioprine, cyclophosphamide, or methotrexate [10]. Other treatments that have been less commonly used with variable success include cranial irradiation and antitumor necrosis factor-alpha therapies [11, 12]. This patient ultimately had no improvement and developed leg swelling after 3 months of steroids, and the patient, his family, and the care team agreed to not pursue further treatment given his age and comorbidities.

## 4. Conclusion

In conclusion, isolated neurosarcoidosis comprises a rare subset of neurosarcoidosis and remains challenging to diagnose and treat. Clinical presentation is variable, with our patient specifically developing weakness and gait disequilibrium. While his granulomatous lesions were first detected in 2008, the lack of reliable confirmatory testing aside from biopsy delayed final diagnosis by 12 years. Finally, while medical and neurological comorbidities complicated the patient's clinical symptoms, the patient notably had no symptomatic improvement on steroids.

## Data Availability

The data that support the findings of this study are available from the corresponding author upon reasonable request.
